# Biofeedback-based training for stress management in daily hassles: an intervention study

**DOI:** 10.1002/brb3.241

**Published:** 2014-06-15

**Authors:** Yuka Kotozaki, Hikaru Takeuchi, Atsushi Sekiguchi, Yuki Yamamoto, Takamitsu Shinada, Tsuyoshi Araki, Kei Takahashi, Yasuyuki Taki, Takeshi Ogino, Masashi Kiguchi, Ryuta Kawashima

**Affiliations:** 1 Smart Ageing International Research Center, Institute of Development, Aging and Cancer, Tohoku UniversitySendai, Japan; 2 Division of Developmental Cognitive Neuroscience, Institute of Development, Aging and Cancer, Tohoku UniversitySendai, Japan; 3 Department of Functional Brain Imaging, Institute of Development, Aging and Cancer, Tohoku UniversitySendai, Japan; 4 Department of Community Medical Supports, Tohoku Medical Megabank Organization, Tohoku UniversitySendai, Japan; 5 Department of Radiology and Nuclear Medicine, Institute of Development, Aging and Cancer, Tohoku UniversitySendai, Japan; 6 Hitachi Ltd.Tokyo, Japan; 7 Central Research Laboratory, Hitachi, Ltd.Saitama, Japan

**Keywords:** Biofeedback training, daily hassles, hippocampus, orbitofrontal cortex, subgenual anterior cingulate cortex

## Abstract

**Background:**

The day-to-day causes of stress are called daily hassles. Daily hassles are correlated with ill health. Biofeedback (BF) is one of the tools used for acquiring stress-coping skills. However, the anatomical correlates of the effects of BF with long training periods remain unclear. In this study, we aimed to investigate this.

**Methods:**

Participants were assigned randomly to two groups: the intervention group and the control group. Participants in the intervention group performed a biofeedback training (BFT) task (a combination task for heart rate and cerebral blood flow control) every day, for about 5 min once a day. The study outcomes included MRI, psychological tests (e.g., Positive and Negative Affect Schedule, Center for Epidemiologic Studies Depression Scale, and Brief Job Stress Questionnaire), and a stress marker (salivary cortisol levels) before (day 0) and after (day 28) the intervention.

**Results:**

We observed significant improvements in the psychological test scores and salivary cortisol levels in the intervention group compared to the control group. Furthermore, voxel-based morphometric analysis revealed that compared to the control group, the intervention group had significantly increased regional gray matter (GM) volume in the right lateral orbitofrontal cortex, which is an anatomical cluster that includes mainly the left hippocampus, and the left subgenual anterior cingulate cortex. The GM regions are associated with the stress response, and, in general, these regions seem to be the most sensitive to the detrimental effects of stress.

**Conclusions:**

Our findings suggest that our BFT is effective against the GM structures vulnerable to stress.

## Introduction

The day-to-day causes of stress, such as misplacing car keys, traffic jams, and minor arguments with family and coworkers, are called daily hassles (Kanner et al. 1981). Daily hassles differ from major life events (Lazarus and Folkman 1984). Many previous studies reported that daily hassles are correlated with stress (Kanner et al. 1981; DeLongis et al. 1988; Bouteyre et al. 2007). We lose the ability to cope effectively after long-term exposure to daily hassles or stress. Although some of these hassles can be dealt with easily, the occurrence of many hassles in quick succession rapidly erodes our ability to cope. Previous studies suggested that daily hassles are correlated with ill health and have identified several neural substrates that are related to stress and negative emotions in daily life (Lazarus and Folkman 1984; DeLongis et al. 1988; Segal and VanderVoort 1993). Previous studies also suggested that activity in brain regions such as the rostrolateral prefrontal cortex (RLPFC) and frontopolar cortex are related to interoceptive awareness (Fleming et al. 2010; McCaig et al. 2011), and that the frontopolar PFC has the potential to be linked preferentially to the meta-awareness of one's own emotional states (Lane et al. 1997; Ochsner et al. 2004). Other reports have shown that interoceptive awareness and the RLPFC/frontopolar cortex are key elements in emotion regulation (Sze et al. 2010). Conversely, negative emotions have been shown to influence the functioning of the RLPFC/frontopolar cortex, and anxiety disrupts the higher order cognitive functions subserved by the RLPFC/frontopolar cortex (Takizawa et al. 2013). Other studies have shown that the orbitofrontal cortex (OFC) is a trigger site for emotions (Bechara and Naqvi 2004; Burgdorf and Panksepp 2006) and is related to interoceptive awareness (Craig 2002; Critchley et al. 2004). Moreover, OFC dysfunction is associated with depression (Bremner et al. 2002; Drevets 2007), anxiety disorder (Milad and Rauch 2007), and posttraumatic stress disorder (PTSD) (Bremner 2002). The subgenual anterior cingulate cortex (sgACC) is also related to mood disorder (Drevets et al. 2008) and PTSD (Herringa et al. 2012) and plays an important role in emotion regulation (Milad et al. 2007). In addition, the hippocampus is vulnerable to stress (McEwen 1999; Fanselow and Dong 2010), and the effect of excessive stress on the brain has been shown to influence the hypothalamic–pituitary–adrenal (HPA) axis and be accompanied by increased blood cortisol levels and neuronal destruction. Excessive secretion of cortisol leads to atrophy of the hippocampus and has a potentially detrimental effect on various psychosomatic parameters (Stokes 1995). When people feel excessive stress, the adaptive response provided by endocrine secretion and immune system is integrated and processed by the brain and becomes apparent as a change in autonomic function and emotion.

Another study suggested that a lack of interoceptive awareness is associated with both compulsive and impulsive self-injurious behaviors (Favaro and Santonastaso 1998). Interoception is defined as the sense of the physiological condition of the body, such as conscious awareness of the emotional processes and behavior related to afferent physiological information arising from the body (Vaitl 1996; Craig 2002). The physiological mechanisms that act as interoceptive stimuli comprise proprioceptive and visceroceptive processes, such as the heart rate (HR) (Domschke et al. 2010). Moreover, although a previous study suggested that interoceptive sensitivity is associated with the pathogenesis of anxiety and anxiety disorders. On the basis of these previous studies, we focused on biofeedback (BF), which promotes the individual's awareness and ability to control internal states, as a means to improve daily hassles.

BF, which is an intervention that involves measuring a person's quantifiable biological signals and conveying the information to the person in real time, is a useful method to provide guidance and reinforcement for the successful management of the physiological response to stress. The main quantifiable biological signals that are related to BF tasks include cerebral blood flow (CBF) and HR (Christopher deCharms et al. 2005). Currently, the BF task is commonly used to improve health, performance, and the physiological changes that often occur in conjunction with changes in thoughts, emotions, and behavior in conditions such as attention deficit/hyperactivity disorder (ADHD) (Alhambra et al. 1995; Boyd and Campbell 1998). We decided to investigate the effectiveness of BF using CBF and HR as an easy and accessible method for prolonged use to reduce the stress associated with daily hassles. We hypothesized that CBF and HR control by performing BF will reduce negative feelings (including anxiety) and stress. Regarding the effect of BF, a previous study suggested that the learning and acquisition of an original relevant strategy that used BF information are important for promoting the effect of BF (Wells 1973). Moreover, as imaging study that used electrodermal activity as a biosignal during the BFT demonstrated increased activation of the anterior cingulate to ventromedial prefrontal cortex and of the cerebellar vermis during the combination of BF and relaxation (Critchley et al. 2001). Another study reported that it is possible to learn RLPFC activity through real-time functional magnetic resonance imaging (rtfMRI) training using a metacognitive awareness strategy (McCaig et al. 2011). Other data also suggest that sgACC activity can be controlled using rtfMRI neurofeedback (Hamilton et al. 2011). Previous rtfMRI study that used rostral ACC activity as a biosignal showed that successful regulation of the rostral ACC yielded pain relief (Christopher deCharms et al. 2005). However, the anatomical correlates of the effects of BF with long training periods remain unclear.

The objective of this study was to investigate the anatomical correlates of BF effects and the psychological changes associated with those effects. Here, we developed a BF system that uses the portable (small sized) near infrared spectroscopy (NIRS) system and selected CBF from the RLPFC to frontopolar cortex and HR as the biological signals associated with BF. We hypothesized that learning to regulate the activity of the RLPFC/frontopolar cortex, which is involved in the introspective evaluation of thought processes, would help change negative mood and negative thought patterns of individuals and enhance stress-coping skills (e.g., emotion regression), by learning to regulate the access to internal states. In addition, we considered that this modality would be easier to perform than real-time functional magnetic resonance imaging (rtfMRI), because our BFT can be performed using the CBF and HR biosignals acquired by NIRS, without having to use an MRI scanner. HR is strongly related to both emotions (Murakami and Ohira 2007; Kreibig 2010) and interoceptive awareness (Pollatos et al. 2007). Interoceptive awareness plays an important role in emotions (James 1884; Katkin 1985; Damasio et al. 2000; Craig 2002). Therefore, we propose that training to control HR will improve negative emotions and enhance activity of brain areas related to emotions, such as the sgACC and pregenual anterior cingulate cortex. We also assessed regional gray matter variation (rGMV) from pre- to postintervention stages of the BFT using voxel-based morphometry (VBM) (Ghaziri et al. 2013).

On the basis of the activation studies of the effects of BF mentioned above, we hypothesize that the brain region from the RLPFC to frontopolar cortical area, which is associated with the awareness of internal states, and the subgenual cingulate to ventromedial prefrontal cortex, which is associated with the control of emotions. We also hypothesize that the volume of the hippocampus increases with improved stress because this area is associated with stress.

## Materials and Methods

### Participants

**Figure 1:**
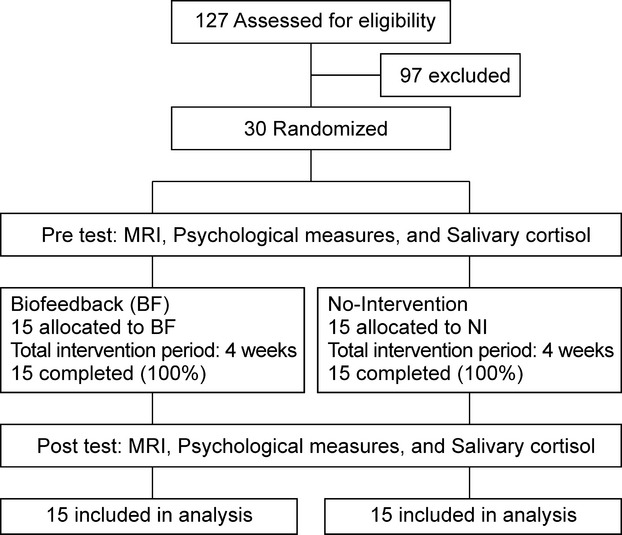
Flow chart of the study.

The study was a randomized, double-blind, controlled, crossover trial that was registered at the University Hospital Medical Information Network Clinical Trials Registry (UMIN000002517). Figure 1 presents a flow chart of the study. In total, 127 participants were recruited in April 2012 through the placement of a leaflet in the local newspaper. Before inclusion, participants were screened using a questionnaire. On the 127 participants, 97 participants were excluded either voluntarily or based on the selection criteria (i.e., a past or present history of any malignant tumor, head trauma, cerebrovascular disease, epilepsy, or any psychiatric disorder). Finally, 30 men were included in this study, which was performed between June 2012 and November 2012. All participants (age range, 23–53 years) were right-handed working native Japanese speakers who had no serious mental disorder. Written informed consent was obtained from each subject in accordance with the Declaration of Helsinki (1991). This study was approved by the Ethics Committee of the Tohoku University School of Medicine. The participants were assigned to two groups, the intervention group (BF group) and the control group (no intervention [NI] group), via a random draw using a computer. The BF group attended BF intervention sessions for 4 weeks. The NI group did not undergo BF intervention and engaged in regular life over the 4 weeks. All participants underwent MRI, psychological measurements, and salivary cortisol evaluation, both on the first day and at 4 weeks after the start of the intervention.

### BF intervention

**Figure 2:**
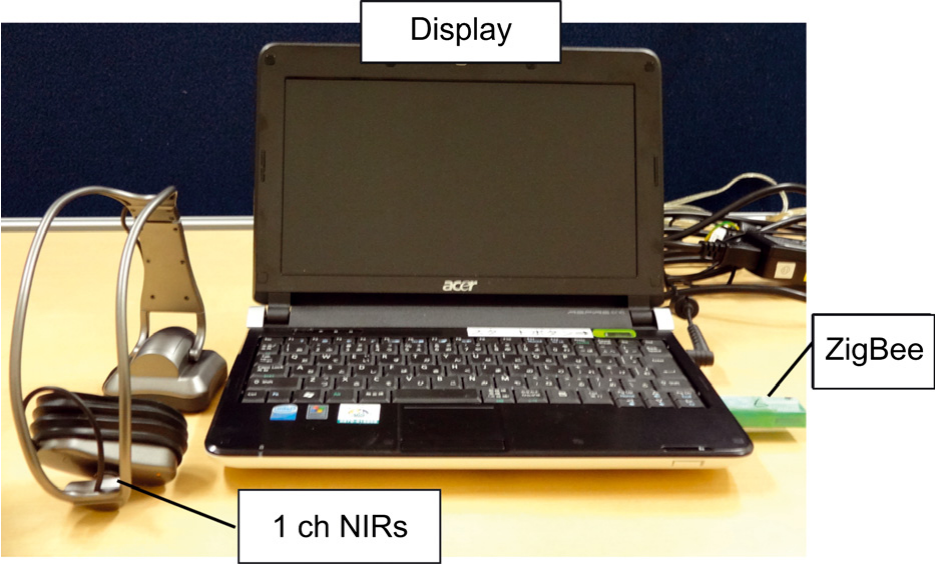
Device used for biofeedback training. Subjects in the intervention group performed a biofeedback-training task using this device.

Participants in the intervention group performed a BFT (combination task for CBF and HR by using our newly developed wearable 1-channel NIRS (1chNIRs) apparatus every day (Fig. 2). The 1chNIRs uses 810-nm near-infrared light that is isosbestic point of oxygenicity hemoglobin and deoxygenation hemoglobin to measure both oxy- and deoxyhemoglobin concentration in brain tissues, as well as the heart rate. Its sampling rate is 10 Hz. The basic program of our biofeedback training uses the original application that made to realize the feedback mentioned above in 1chNIRs. Our BFT is two kinds of CBF control and the HR control in the frontal pole. The BFT does not control it at the same time and become the game constitution to control separately each. We used 8.9 inches of WSVGA (1024 × 600) liquid crystal displays of the deployment for Aspire one AOA150 to operate the application mentioned above. Additionally, 1chNIRs transmits data to a personal computer (PC), in which the BFT task is managed using the ZigBee protocol.

The signals of CBF and HR were calculated using the information of the blood flow obtained from the photo diode (PD) 1 cm and PD 3 cm. The CBF was measured by 2PD. This method is capable of detecting only the changes in blood flow due to brain activity minus the influence of skin blood flow. We define cerebral blood flow by following the formula from *y* and *x* of formulae 1999, the regression line *α* calculated during calibration, and HR.


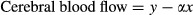
(1.1)

The HR detected was pulse wave data of the skin blood flow obtained from PD 1 cm. Then, we calculated the HR corresponding to each pulse of once (times/second). We used as the point at which blood flow value data are the minimum value of the separated pulse wave. The following points can be given as reasons. Because the waveform of the blood flow value shows the most change when taking a minimum value, it can be expected the most accurate. Minimum value is the minimum of data among the five most recent data. It was not valid if the distance between the minimum value is out of range of the pulse from the standard (48–180 times/min) for noise rejection, and the previous value is retained. We set a point in time when a bloodstream level took a local minimum with *t*_*i*_(*i* ≥ 0) and we defined HR as follows.


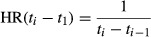
(1.2)

However, *t*_1_ of the time because started, assumed it *i* > 0.

About the analysis of two kinds of signals (CBF and HR) provided from 1ch NIRs, a result of having examined a method to remove influence of the skin blood flow, we established PD which measured only skin blood flow separately and thought that supplementary plus could calculate CBF quantity using a value of provided skin blood flow. The light detected in PD 1 cm is influenced only by the hemoglobin density of skin. Therefore, it becomes like the following expression.


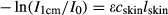
(1.3)

3 cm PD also includes information of both the hemoglobin concentration of CBF and the skin blood flow.


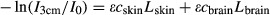
(1.4)

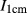
: intensity of the light detected by the 1 cm PD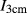: intensity of the light detected by the 3 cm PD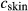: hemoglobin concentration of skin blood flow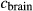: hemoglobin concentration of cerebral blood flow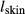: effective optical path length of the light detected by the 1 cm PD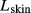: effective optical path length in the skin of the light detected by 3 cm PD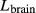: effective optical path length in the brain of the light detected by 3 cm PD
ε: isosbestic coefficient

By the formulae 2000 and 2004,


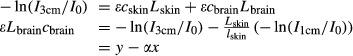
(1.5)


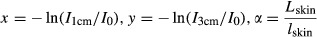


By the above formulae, it is possible to obtain only the concentration of hemoglobin in CBF (*c*_brain_) by removing the influence of skin blood flow. We provide a procedure for calibration of determining *α* by calculating a regression line of *y* = *αx* + *β* from the data of the rest of the participants. By the method described above, we were able to remove the influence of skin blood flow. Therefore, we were used the 1LED/2PD method.

**Figure 3:**
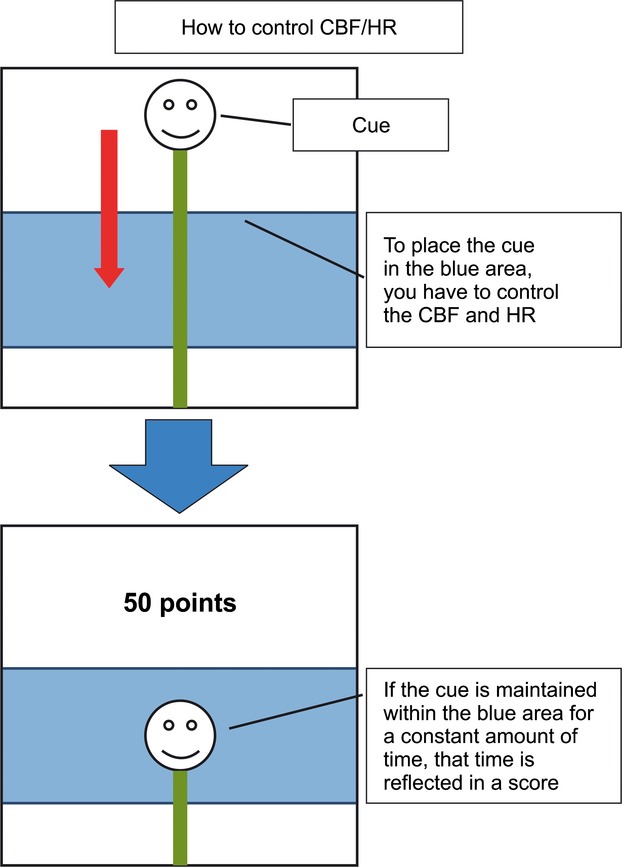
Screenshots of the biofeedback-training task The biofeedback-training task aims to maintain cerebral blood flow (CBF) and heart rate (HR) within the designated range by controlling them separately or simultaneously. The blue line differed in width according to training level. Participants were asked to ensure that visual cue was kept inside the blue line for a given length of time and were trained to control a set of the BFT.

The BFT was designed to control either regional CBF (rCBF), which is calculated from total hemoglobin concentration, or HR. The participants were asked to monitor their own rCBF in frontopolar cortex or HR on a PC display, and were ordered to increase or decrease them according to instructions from a PC. For example, the message “to control the CBF” or “to control the HR” is displayed on a PC display and the blue line is disposed above or below on a PC display. We managed it by programming where a blue line appears. The cue appears at the same time. The cue which they see is a biosignal of their own rCBF in frontopolar cortex or HR. The cue moves up and down by their biosignal. The training was conducted as 10 sets, of 120 sec each, per day. The participants see a cue during the BFT. In the case of both CBF and HR, the participants move a cue within the range of a blue line. To place the cue in the blue area, you have to control the CBF and HR. If the cue is maintained within the blue area for a constant amount of time that time is reflected in a score (Fig. 3). If the cue does not reach the target range after 10 sec from a training start, an advice message is displayed on a display. If the cue achieves the target range within 10 sec, level is increased and difficulty of training also increases. The training moved on to the next set when the training was uninterruptedly maintained for 15 sec within a target range. At the end of each set, a score was calculated using the formulas shown below, based on remaining time and time over which CBF and HR were maintained within the target values. If target retention was accomplishing for 15 sec within the time limit, the following formula was used: score = {(remaining time [sec] + 15 sec) + (maintaining time [sec] within the target range − 15 sec × 0.5)}/120 sec × 100. If target retention was not accomplished for 15 sec within the time limit, the following formula was used: score = (maintaining time [sec] within the target range × 0.5)/120 sec × 100.

In addition, user level (difficulty) was calculated from the average score of 100 previous sets. If the average score ranged from 0 to 9 points, user level was set as 1. If the number of times training is less than 100 sets, it is calculated as 0 points. In the case of Figure 3, a low score is shown because target retention was not accomplished for 15 sec within the time limit. Conversely, a perfect score is displayed if target retention was accomplished for 15 sec within the time limit. This training was mechanism that it proceeded to the next training level if the cue stay 15 sec within the target range continuously. In fact, many participants did not take 120 sec at training time and the training time per day of participants was approximately 5 min. Participants assigned to the BF group performed the BFT in their homes. The training time of the BFT was about 5 min once a day, in accordance with a previous study (Keefe and Gardner 1979; Freedman and Ianni 1983). In this study, there was not the dropout.

### Experimental procedure

Participants underwent the examinations at separate time points: before the intervention and after the intervention. All participants underwent brain MRI, completed psychological measures, and provided a saliva sample in a university laboratory. After about 35 min into the MRI scan, the participants underwent psychological tests for about 120 min. Participants provided a saliva sample at 4 pm, which required about 5 min. The total experimental time of each examination was 245 min.

### Psychological measures

To determine the effect of our BFT on daily hassles, we used the following psychological measures as pre- and postintervention evaluations. We selected the evaluation measures based on the facts that daily hassles are related to depression (Jung 1989), negative mood, and ill health (DeLongis et al. 1988). Moreover, job stress was included as a cause of daily hassles (Lazarus and Folkman 1984). The measures used were (1) the Center for Epidemiologic Studies Depression Scale (CES-D), which measures the respondent's level of depression symptoms within the past week (Radloff 1977; Shima 1985); (2) the General Health Questionnaire 30, which measures psychological distress (Goldberg 1972; Nakagawa and Daibo 1985); (3) the Positive and Negative Affect Schedule (PANAS), which measures positive and negative affects as states (Watson et al. 1988; Sato 2001); and (4) the Brief Job Stress Questionnaire (BJSQ), which measures job stress (Shimomitsu 2004). The BJSQ comprises 57 items and measures three domains: work stressors, stress response, and modified factors. The work stressors and stress response domains of the BJSQ were used in this study.

### Saliva sampling

We collected saliva samples to measure cortisol levels. Distressing psychological stimuli are associated with an increased cortisol level (Fukuda and Morimoto 2001). Taking into consideration the participants' circadian cortisol rhythms, we collected all saliva samples at 4:00 pm on weekdays, both before and after the intervention. We selected this time because people are less affected by circadian cortisol rhythms at this time of day (Riad-Fahmy et al. 1983). Participants refrained from drinking, eating (Toda et al. 2004), and exercise (O'Connor and Corrigan 1987) for 2 h before saliva sampling. Saliva samples were collected using the salivette apparatus (Sarstedt, Nümbrecht, Germany).

### Measurement of salivary cortisol levels

To assess physiological stress, we used a technique to measure salivary cortisol reported by a previous study (Kotozaki and Kawashima 2012). Saliva samples were centrifuged at 3000 rpm for 5 min, and we stored the supernatant solutions in airtight containers at −80°C and measured salivary cortisol in these solutions using semi-microcolumn high-performance liquid chromatography (HPLC) system (Shiseido, Tokyo, Japan). The following conditions were used for HPLC analysis: the mobile phase used for preprocessing was a 5 mmol/L phosphoric acid buffer solution (pH = 6.9) and acetonitrile at a ratio of 98:2, which flowed through the columns at a rate of 1 mL/min; and the mobile phase was a 10 mmol/L phosphoric acid buffer solution (pH = 6.9) and acetonitrile at a ratio of 78:22, which flowed through the columns at a rate of 0.1 mL/min. The column temperature was maintained at 35°C, and the detection wavelength was 242 nm.

### Image acquisition

All MRI data were acquired with a 3T Intera Achieva MRI scanner (Achieva, Philips, Best, the Netherlands) at Tohoku University. Using a magnetization-prepared rapid gradient echo sequence, high-resolution T1-weighted structural images (240 × 240 matrix, repetition time = 6.5 msec, echo time = 3 msec, field of view = 24 cm, 162 slices, 1.0 mm slice thickness) were collected.

### Voxel-based morphometric analysis

Voxel-based morphometry (VBM) was used to investigate the morphological changes in the participants' brains. Preprocessing of morphological data was performed using the VBM2 software package (Gaser 2007), which is an extension of SPM2. To reduce scanner-specific biases, first we used a customized gray matter (GM) anatomical template that was based on prior probability maps of gray and white matter images created from T1-weighted structural imaging data that were acquired from all participants before the intervention. Subsequently, the T1-weighted structural imaging data of participants were segmented into gray and white matter partitions using the participants' gray and white matter prior probability maps. The resulting images included gray and white matter partitions in the native space of the scanner. The GM probability map generated from subject data was then used to create a new GM-partitioned standard image; that is, the normalization parameters determined by our segmentations were then applied to the native T1-weighted structural image. These normalized, T1-weighted structural data were then segmented into gray and white matter partitions. To facilitate optimal segmentation, we estimated normalization parameters using an optimized protocol (Good et al. 2001). Moreover, we performed a correction for volume changes (modulation) by modulating each voxel with the Jacobian determinants derived from the spatial normalization; this also allowed us to test for regional differences in the absolute amount of GM (Ashburner and Friston 2000). All images were subsequently subjected to 12-mm Gaussian smoothing. Finally, the change in rGMV between pre- and postintervention images was computed at each voxel for each participant. We only included voxels that showed GMV probabilities >0.10 in both pre- and postscans in these computations, to avoid possible partial volume effects at the borders between GM and white matter, as well as between GM and CSF. The resulting maps representing the rGMV before intervention and the rGMV change between the pre- and postintervention scans (pre–post) were then used in the group-level analysis described below.

We think the methodology in our original manuscript using VBM2 is well established, because many papers have been published using this method (Ilg et al. 2008), as well as our previous ones (Takeuchi et al. 2010, 2011, 2012, 2013a,b, 2014). These procedures (preprocessing in VBM2 and statistical analyses in SPM/VBM of different versions) were also followed in these previous studies. So, our original results are also as trustworthy as these previous studies.

Actually, we have usually adopted VBM2 instead of VBM5 or VBM8 for the preprocessing of T1-weighted structural imaging data so far, because of the following reasons. The T1-weighted images obtained using the MPRAGE sequence of our Philips scanner were incompatible with preprocessing of more recent versions, such as VBM5/SPM5 and VBM8/SPM8. This is because use of VBM5 or SPM5 results in many apparent segmentation errors, unlike use of the VBM2 optimized protocol. These segmentation errors that are apparent from a first glance did not exist when VBM2 was used. Furthermore, use of DARTEL/VBM8 and SPM8 did not result in apparent segmentation errors that are apparent from a first glance at least. However, the test–retest reliability of total gray matter volume of between pre- and postearthquake images in the 42 subjects in our current study was 0.632 (compared to 0.947, when VBM2 was used).

In addition, in accordance with your suggestion, we have conducted preprocessing by using DARTEL/SPM8, as well. In this case, the test–retest reliability of average of the gray matter intensity between pre- and postearthquake images in the 42 subjects was 0.746 (compared to 0.955, when VBM2 was used). We also suspect something incompatible with DARTEL/SPM8. These findings do not indicate VBM5/VBM8/DARTEL preprocessing is worse, but do indicate the compatibility between the T1-weighted structural images of certain sequences and VBM5/VBM8/DARTEL. From the above reason, VBM5/VBM8's preprocessing is not compatible with our T1-weighted structural images, and we cannot alter this.

### Statistical analyses

The psychological and salivary data were analyzed using the PASW statistical software package (ver. 18 for Windows; SPSS Inc., Chicago, IL). Demographic and clinical data were subjected to one-way analyses of variance. One-way analyses of covariance were conducted by including the differences between the pre- and postintervention scores as dependent variables, and pretest scores as covariates of each psychological measurement. Because our primary endpoint was the beneficial effect of intervention training, test–retest changes were compared between the BF and NI groups using Bonferroni post hoc test for significance, *P* < 0.05, one-tailed. In the group-level analysis of the rGMV, we examined the group-wise differences in rGMV changes using the factorial design option of SPM5. The effect of the intervention was estimated by comparing the changes between the pre- and postintervention measurements as described above, followed by a comparison between groups at each voxel using age and total GMV before intervention as covariates. The data were corrected for multiple comparisons across the whole brain at the nonisotropic adjusted cluster level (Hayasaka et al. 2004), with an underlying voxel-level threshold of *P* < 0.0025. Nonisotropic adjusted cluster-size tests should be applied when data are nonstationary (i.e., not uniformly smooth), as are VBM data (Hayasaka et al. 2004).

In addition, we conducted a separate analysis for the ventromedial PFC and sgACC using the small volume correction (SVC) implemented in SPM5. In view of our a priori hypothesis regarding RLPFC and sgACC changes, we applied SVC using a sphere threshold (10-mm radius) at a family-wise error corrected *P* < 0.05, thereby restricting the search volume and increasing sensitivity. The regions of interest (ROI) for the ventromedial PFC and sgACC were defined using masks created by WFU PickAtlas (Maldjian et al. 2003). The coordinate for the RLPFC was taken from the main effect peak reported in the study of McCaig et al. (MNI coordinates: *x* = −16, *y* = 62, *z* = 16) (McCaig et al. 2011). The peak of this area represented activation throughout the RLPFC, which plays a role in the meta-cognitive awareness regulation strategy (McCaig et al. 2011). The coordinate for the sgACC was taken from the main effect peak reported in the study of Herringa et al. (2012) (MNI coordinates: *x* = 6, *y* = −4, *z* = −8). This peak represented cluster peak coordinates of the sgACC, which are inversely related to the Clinician-Administered PTSD Scale scores used for the evaluation of posttraumatic stress symptoms (Herringa et al. 2012).

## Results

### Psychological measures

The demographic and clinical data of the study participants are given in Table 1. The subject age and years of service in each company did not differ significantly between the BF and NI groups. Comparisons of psychological changes before and after intervention between the groups are shown in Table 2. The BF group exhibited a significant decrease in the postintervention CES-D (*F*_1,27_ = 4.36, *P* < 0.05) and PANAS-NA scores (*F*_1,27_ = 5.75, *P* < 0.05). The BF group also showed a significant decrease in the BJSQ tension scores *F*_1,27_ = 6.83, *P* < 0.05, the BJSQ depression scores (*F*_1,27_ = 4.32, *P* < 0.05), and the BJSQ stressors of working environment scores (*F*_1,27_ = 11.68, *P* < 0.01) as well as a significant increase in the BSJQ aptitude for the job scores (*F*_1,27_ = 7.46, *P* < 0.05).

**Table 1:** Baseline demographic and clinical data of the study subjects

	BF group (*n* = 15)		NI group (*n* = 15)		
			
Factor	Mean	SD	Mean	SD	*P*^1^
Age (years)	41.7	7.7	42.9	8.1	0.680
Years of service	19.5	7.5	22.1	6.8	0.329

BF, biofeedback; NI, no intervention; SD, standard deviation.

1 One-way analysis of variance.

**Table 2:** Psychological test scores and salivary cortisol levels before and after biofeedback training

	BF group				NI group					
				
	Pre		Post		Pre		Post			
						
Measures	Mean	SD	Mean	SD	Mean	SD	Mean	SD	Planned contrast	*P*^1^
CES-D score	8.13	6.62	7.07	6.87	11.07	6.91	14.73	12.65	BF < NI	0.039*
PANAS-PA score	27.13	5.07	26.87	5.74	20.33	7.24	22.07	8.22	BF > NI	0.323
PANAS-NA score	19.20	7.58	16.87	7.21	16.47	7.21	18.60	7.59	BF < NI	0.020*
BJSQ psychological work load (quantity) score	2.80	1.37	2.73	1.39	2.60	1.35	2.73	1.03	BF < NI	0.312
BJSQ psychological work load (quality) score	3.00	0.76	2.93	0.80	2.60	1.18	2.53	0.99	BF < NI	0.300
BJSQ physical work load score	2.80	1.01	2.80	1.01	2.80	1.21	2.60	1.06	BF < NI	0.220
BJSQ stressors of personal relations in a workplace score	3.00	1.00	3.07	1.16	2.80	1.21	2.80	0.86	BF < NI	0.309
BJSQ stressors of working environment score	2.73	1.16	1.67	1.40	2.60	1.18	2.93	1.22	BF < NI	0.001**
BJSQ job control score	3.60	0.99	3.67	0.98	3.27	1.03	3.47	0.83	BF > NI	0.475
BJSQ skill utilization score	3.47	0.64	3.33	0.72	3.27	0.80	2.87	0.83	BF > NI	0.060
BJSQ aptitude for job score	2.87	0.99	3.53	1.36	3.33	1.35	3.20	1.32	BF > NI	0.020*
BJSQ worth working score	2.60	1.18	2.73	1.33	3.27	1.44	3.20	1.08	BF > NI	0.433
BJSQ vigor score	3.20	0.77	3.40	0.74	2.73	1.22	2.73	1.28	BF > NI	0.101
BJSQ tension score	3.27	1.22	2.40	1.18	3.13	1.06	3.13	1.13	BF < NI	0.008*
BJSQ fatigue score	3.27	1.10	3.27	1.10	2.93	1.33	2.47	0.91	BF < NI	0.023*
BJSQ anxiety score	3.00	1.20	3.13	1.41	3.13	0.92	3.20	1.08	BF < NI	0.446
BJSQ depression score	3.73	1.39	2.87	1.25	3.27	1.10	3.27	1.16	BF < NI	0.039*
BJSQ somatic stress response score	3.27	1.33	3.20	1.32	3.33	1.05	3.33	1.29	BF < NI	0.340
Salivary cortisol level	4.14	3.48	2.18	2.69	4.04	4.52	5.44	5.05	BF < NI	0.008**

Bold indicates significantly different values.

BF, biofeedback; NI, no intervention; SD, standard deviation; CES-D, Center for Epidemiologic Studies Depression Scale; PANAS-PA, Positive and Negative Affect Schedule-Positive Affect; PANAS-NA, Positive and Negative Affect Schedule-Negative Affect; BJSQ, Brief Job Stress Questionnaire.

1 One-way analyses of covariance using pre-/postintervention differences in psychological measures as dependent variables and preintervention scores as covariates (one tailed).

* Significant at *P* < 0.05 level after Bonferroni correction.

** *P* < 0.01.

### Salivary cortisol levels

**Figure 4:**
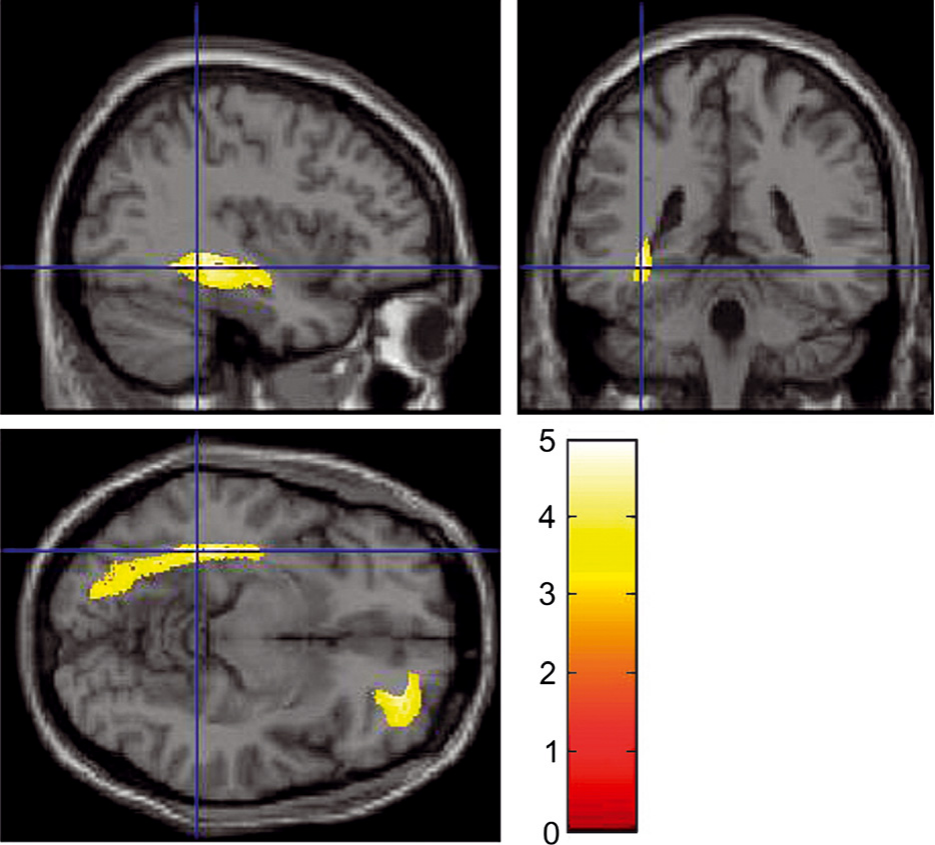
The regional gray matter volume of the right lateral orbitofrontal cortex and an anatomical cluster that included mainly the left hippocampus increased in the BF group compared to the NI group. Results are shown at a significance level of *P <* 0.05, corrected for multiple comparisons at the cluster level with an underlying voxel level of *P <* 0.0025. The color density represents the T score.

The results of comparisons between the salivary cortisol levels measured before and after intervention are shown in Table 2. The BF group exhibited a significant decrease in salivary cortisol levels (*F*_1,27_ = 5.53, *P* < 0.05), indicating a greater reduction in stress in this group compared to the NI group (Fig. 4).

### Effects of BF intervention on gray matter structures

**Figure 5:**
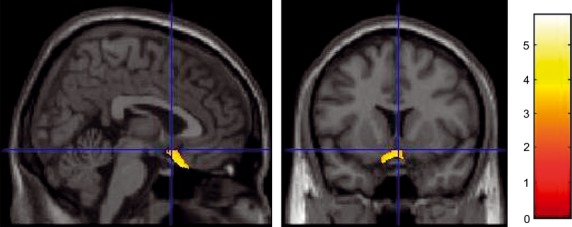
Another area (the left subgenual anterior cingulate cortex) showing an increase in the regional gray matter volume in the BF group. Results are shown for *P* = 0.0001 corrected for visualization purposes. Note that the cluster may look small compared with the extent of the significant correlation described in the Results section because of the thresholds used in the SVC.

Compared with the NI group, as shown in Figure 2, the BF group showed a significant increase in the rGMV of the right lateral OFC, which included the RLPFC (MNI coordinates: *x* = 30, *y* = 36, *z* = −13; *t* = 4.98; *P* = 0.013, corrected for multiple comparisons at the nonisotropic adjusted cluster level with an uncorrected cluster-determining threshold of *P* < 0.0025), and the anatomical cluster that included mainly the left hippocampus and parahippocampal gyrus, but also extended into the fusiform and visual cortices (MNI coordinates: *x* = −37, *y* = −38, *z* = −5; *t* = 4.56; *P* < 0.001, corrected for multiple comparisons at the nonisotropic adjusted cluster level with an uncorrected cluster-determining threshold of *P* < 0.0025). In addition, the BF group showed an increase in the rGMV around the left sgACC (MNI coordinates: *x* = −7, *y* = 20, *z* = −28; *t* = 5.87; SVC for areas with a strong a priori hypothesis: *P* = 0.0001 corrected for FWE at the voxel level within the sgACC). The SPM contrast used was: NI group (rGMV_pre_ – rGMV_post_) – BF group (rGMV_pre_ – rGMV_post_). No other significant results were found in this analysis (Fig. 5).

## Discussion

The objective of this study was to investigate the anatomical correlates of BF effects, as well as the psychological changes associated with these effects. The present study revealed that BF had effects on the rGMV, psychological test scores, and salivary cortisol levels associated with daily hassles. These results are consistent with our hypothesis that BF reduces daily stress; increases the GMV of the OFC, sgACC, and hippocampus (related to stress); and decreases salivary stress-marker levels, as discussed in detail below.

Regarding changes in brain structures, this study revealed that after 4 weeks of BF intervention, the rGMV was increased in the right lateral OFC, including the RLPFC, which is involved in the action of interoceptive awareness (Craig 2002; Critchley et al. 2004) and emotion regulation (Milad et al. 2007), in the left sgACC, which plays a key role in emotion control (Milad et al. 2007), and in the left hippocampus, the volume of which is vulnerable to stress. Previous studies have suggested that the OFC is associated with stress and is responsible for depression, anxiety disorders, and PTSD (Bremner et al. 2002; Drevets 2007; Hakamata et al. 2007; Milad and Rauch 2007; Sekiguchi et al. 2013). The OFC has an important role in the control of negative emotions, such as anger and sadness, whereas the amygdala and anterior cingulate cortex are linked to stress response (Blair et al. 1999). The OFC plays a crucial role in emotion regulation and response control, during which it is thought to downregulate the activity of the amygdala (Quirk and Beer 2006). A previous study has suggested that the OFC participates in the executive control of information processing and behavioral expression by inhibiting the neural activity associated with negative events or experiences (Shimamura 2000). That study showed that people with psychiatric disease and people who experienced intense fear have decreased perfusion in the OFC, or decreased GMV. Conversely, another study showed that it is possible to increase the GMV of the right OFC via training, such as meditation (Luders et al. 2009). We assume that the increase in the GMV of the right OFC afforded by our daily BFT may have resulted in an improvement in this area's function, such as emotional regulation and response control. It is considered that the RLPFC, including the OFC, is related to interoceptive awareness (Ochsner and Gross 2005; Fleming et al. 2010; McCaig et al. 2011), thus functioning in self-related processing, such as the monitoring, manipulation, and evaluation of information generated internally (Christoff and Gabrieli 2000; Takeuchi et al. 2013a,b). Moreover, this area may be related to emotion (James 1884; Damasio and Spudis 1996; Herbert et al. 2011), possibly via the association between interoceptive awareness and emotion (Ochsner and Gross 2005; Fleming et al. 2010). We consider that the function of the RLPFC, which is related to interoceptive awareness and emotions (James 1884), was enhanced by our BFT, and that training for interoceptive awareness leads to the simultaneous increase in the rGMV of the RLPFC and improvement of emotional state. Moreover, our BFT resulted in increased GMV of the anatomical cluster that included mainly the left hippocampus. The main functions of the hippocampus are related to memory, learning, and spatial learning function (Broadbent et al. 2004). The hippocampus is also related to stress reactions, such as anxiety (McEwen et al. 2012). A previous animal study has shown that stress affects synaptic plasticity, dendritic morphology, neurotoxicity, and neurogenesis in the hippocampus (Kim and Yoon 1998). Moreover, stress impairs hippocampal-dependent forms of learning, such as spatial memory (Diamond et al. 1996). The stress response is a complex biochemical reaction involving the release of various chemicals, such as glucocorticoids, that modulate learning and memory (McGaugh et al. 1996). Glucocorticoids are secreted according to stress experiences and play many roles in homeostasis (Snyder et al. 2011). The hippocampus contains many glucocorticoid receptors, which render it more vulnerable to long-term stress compared to most other brain areas (Joels 2008; Snyder et al. 2011). This means that glucocorticoids are emitted if the brain feels a strong stress, and these molecules will adversely affect the synaptic plasticity of the hippocampus. Consequently, hippocampal neurons are destroyed, and the hippocampus becomes atrophic. Such atrophy of the hippocampus has been confirmed in people with depression (Bremner et al. 2000) or PTSD symptoms (Bremner et al. 1995; Karl et al. 2006), who experience intense stress. Interestingly, several researchers have suggested that the capacity of the atrophied hippocampus may increase after some types of training. Previous studies of exercise training suggest that hippocampal volume increases after aerobic exercise (Erickson et al. 2011) and juggling training (Boyke et al. 2008). Moreover, GM concentration in the hippocampus is increased by meditation training (Hölzel et al. 2011). In those studies, the training modalities were performed intensively throughout periods such as 8 or 12 weeks. Although the daily training time was short, these protocols provided sufficient training time during this period. It is thought that the brain area that was weakened by stress or anxiety was enhanced by training consciously for a long period. We think that the results of our study reflect the effect reported in previous studies, because our BFT was carried out over a similar training period and for 5 min every day. In addition, the BFT led to increased rGMV in the sgACC. The sgACC controls emotion (James 1884) and changes in the GMV of the sgACC are related to depression (Bremner et al. 2000). Our BFT controlled emotion by regulating sgACC activity. Therefore, we consider that this brain region and brain function plasticity was modulated by the BFT.

In addition, we observed psychological changes after our BFT. Compared to the NI group, the BF group exhibited a significant decrease in the postintervention CES-D, PANAS-NA, and BSJQ-aptitude for job scores, as well as a significant increase in the BJSQ tension, BJSQ depression, and BJSQ stressors of working environment scores. The BFT is used as a tool to improve depression. The investigation about BF effect has been considered. A previous study reported that patients with depression exhibit a significant decrease in depression symptoms after performing a BFT once a week for 4 weeks (Karavidas et al. 2007). Another study demonstrated that patients with different degrees of depression showed a significant decrease in depression and anxiety after performing a BFT over 2 weeks (Siepmann et al. 2008). The BFT is also used as a tool to improve stress conditions, including job-related stress and PTSD. In a previous work, the intervention group had a significantly decreased stress level after 4 weeks of BF intervention for physicians (Lemaire et al. 2011). Moreover, a study of veterans with PTSD showed that heart rate variability (HRV)-BF significantly increased the HRV while reducing the symptoms of PTSD (Tan et al. 2011). The results of these studies may be attributable to enhanced parasympathetic activity by training using HRV-BF (Karavidas et al. 2007; Siepmann et al. 2008; Lemaire et al. 2011; Tan et al. 2011). HRV-BF likely reinforces peripheral HR modulation by arterial baroreceptors, as well as by chemoreceptors and cardiopulmonary mechanoreceptors. The BFT may also enhance vagal HR regulation by evoking focused concentration in combination with emotional self-control. Although the BFT administered in our study used a combination of CBF and HRV, emotional self-control ability may also have been enhanced by this BFT, since self-control of CBF and HRV was practiced every day. Moreover, our results revealed an improvement in job stress, such as tension, depression, and aptitude for the job, after the BFT. Likely, participants became able to deal with job stress in the same way as they dealt with daily stress via enhancement of their self-control ability after the BFT.

This study had several limitations. It is necessary to investigate the effects of the BFT in women because we enrolled only men in this study. The current results alone were not conclusive, and we cannot state with certainty that our BFT is effective in most people.

Furthermore, we observed a significant decrease in salivary cortisol levels in the BF group compared with the NI group. The relationship between stress and cortisol level has been investigated for a long time. A previous study suggested that cortisol responds as a psychological stressor with high intensity (Dickerson and Kemeny 2004). Other previous studies suggested that cortisol is related to chronic stress. It is suggested that people who experience job stress and unemployment have high salivary cortisol levels in the morning or at night (Ockenfels et al. 1995; Steptoe et al. 2000). Several middle-aged people participated in the present study. We think that individuals in this age group are likely to be exposed to job stress, in addition to daily stress, and may have developed damage to some brain areas, such as the OFC and the hippocampus, after enduring a significant amount of stress over a long period. Both the OFC and the hippocampus are part of the stress response circuitry and play an important role in emotion (McEwen and Magarinos 1997; Kim and Yoon 1998; Shimamura 2000). We think that the BFT might have improved OFC and hippocampal functioning, which was weakened due to various stresses. Thus, cortisol secretion decreased, because of enhanced hippocampal function after the BFT. We hope that the BFT developed by our group may serve to relieve stress related to daily hassles, and we expect that our BFT will be useful as a future treatment for disorders such as depression and ADHD.
